# The role of the oral cavity in SARS-CoV-2- and other viral infections

**DOI:** 10.1007/s00784-023-05078-z

**Published:** 2023-06-13

**Authors:** Dieter Hoffmann

**Affiliations:** Institute of Virology, Technische Universität/Helmholtz Zentrum München, Trogerstr. 30, 81675 Munich, Germany

**Keywords:** Oral cavity, Virus, Replication, Droplets, Aerosol, Mouth rinse

## Abstract

**Objective:**

This study aims to review the role of the oral cavity in SARS-CoV-2- and other viral upper respiratory tract infections.

**Material and methods:**

Data reviewed in the text have been researched online and also reflect personal expertise.

**Results:**

Numerous respiratory and other viruses replicate in the oral cavity and are transmitted via aerosols (< 5 µm) and droplets (> 5 µm). SARS-CoV-2 replication has been documented in the upper airways as well as in oral mucosa and salivary glands. These sites are also virus reservoirs that can infect other organs, e.g., the lungs and gastrointestinal tract, as well as other individuals. Laboratory diagnosis of viruses in the oral cavity and upper airways focuses on real-time PCR; antigen tests are less sensitive. For screening and monitoring infections, nasopharyngeal and oral swabs are tested; saliva is a good and more comfortable alternative. Physical means like social distancing or masks have been proven successful to reduce the risk of infection. Both wet-lab and clinical studies confirm that mouth rinses are effective against SARS-CoV-2 and other viruses. Antiviral mouth rinses can inactivate all viruses that replicate in the oral cavity.

**Conclusions:**

The oral cavity plays an important role in viral infections of the upper respiratory tract: it serves as a portal of entry, a site of replication, and a source of infection by droplets and aerosols. Physical means but also antiviral mouth rinses can help reduce the spread of viruses and contribute to infection control.

## Introduction

Severe acute respiratory syndrome coronavirus type 2 (SARS-CoV-2) has caused one of the largest pandemics in human history. This has been possible because of a specific viral feature: its high transmissibility at the pandemic onset end of 2019. During one infection, the virus was passed to 3.6 to 6 others in a susceptible population in a European setting, measured by the basic reproductive number R0 [1]. During the last 3 years, even better transmissible variants have evolved [2]. As a respiratory virus, SARS-CoV-2 spreads through droplets and aerosols, which are emitted during normal breathing, but to a much higher extent during speaking, singing, coughing, and sneezing. As the oral cavity is anatomically and functionally involved in these processes, it is conceivable that it plays a paramount role in SARS-CoV-2 transmission. Thus, a better understanding of the oral cavity as an entry point and reservoir of SARS-CoV-2 and other viruses is critical for infection control. In this review, both epidemiology and wet-lab data will be critically discussed and put into a medical context.

## Viral infections and transmission through the respiratory tract and oral cavity

Respiratory viruses infect susceptible individuals through droplets and aerosols that reach mucous membranes of the upper respiratory tract and oral cavity. Airborne liquid particles > 5 µm are considered droplets, < 5 µm aerosols. Even though size influences the fate of airborne particles, the 5-µm threshold has been defined artificially [3]. Airborne particle size usually decreases by evaporation or can increase by coalescence. Aerosols remain suspended in the air for several hours, particularly when their size decreases quickly in dry air. Larger droplets sink to surfaces in minutes, particularly in humid air [3]. SARS-CoV-2 stays infectious in droplets and aerosols with a half-life of 1 h [4, 5].

Droplets and aerosols expelled during breathing and talking originate mainly from the respiratory tract [6]. During dental procedures, droplets and aerosols naturally emerge from the oral cavity. Thus, preprocedural antiviral mouth rinses are expected to reduce infectious virions in those aerosols.

Aerosols are more likely to be inhaled deeper, thus reaching lower portions of the respiratory tract than larger droplets. The cellular SARS-CoV-2 receptors ACE2 and TMPRSS have been well-characterized and detected in oral mucosa, including the tongue, gingival tissue, and salivary glands [7]. Influenza virus replication was detected in several tissues of ferrets, as soft palate, nasal cavity, and lungs [8]. While other respiratory viruses, as RSV, metapneumo- or parainfluenza viruses replicate mainly in the respiratory epithelia, influenza viruses and SARS-CoV-2 infect multiple tissues, sometimes causing clinical illness in other organs. Examples are myocarditis as a complication of influenza viremia and SARS-CoV-2 infection of endothelial cells, possibly causing thrombosis.

## SARS-CoV-2

As a beta coronavirus, SARS-CoV-2 resembles SARS-CoV that caused an epidemic in Asia in 2003 [9]. The human coronaviruses (hCoV) HKU1 and OC43 also belong to the beta genus and are among the most frequent causes for common colds [10]. Other common cold viruses are types NL63 and 229E, which belong to the alpha genus. All four types are endemic worldwide and are detected with similar frequencies [11]. The SARS-CoV-2 genome comprises around 30,000 nucleotides and encodes numerous proteins [12]. They orchestrate viral replication, including cell entry and egress. The relatively big and complex genome requires a proof-reading polymerase to keep it stable. Even though it reduces the SARS-CoV-2 mutation rate, it is estimated that the virus accumulates 33 genome mutations per year [13]. This is also due to the exceedingly high number of infected subjects, each harboring 10^9^ to 10^11^ virions at the peak of infection [14].

From the upper respiratory tract and oral cavity, SARS-CoV-2 can spread to lower respiratory tissues and other organs, as the gastrointestinal tract or blood vessels.

## SARS-CoV-2 and COVID-19

SARS-CoV-2 is associated with multiple clinical symptoms, summarized as coronavirus disease 2019 (COVID-19). It is antigenically distinct enough from endemic human coronaviruses to render the entire human population susceptible, thus causing one of the largest pandemics ever. Several features are characteristic for SARS-CoV-2 compared to other respiratory viruses:

- Pandemic spread with prevalence varying in waves.
- New variants emerging and replacing previous ones on a global level.
- Replication in gingival tissue and salivary glands has been documented.
- Various cell types can be infected in cell culture, facilitating systemic infection.
- Sequelae in various organs, e.g., the blood vessels, lungs, heart, kidneys.
- Generalized symptomatic complications as multisystem inflammatory syndrome and long COVID-19.

Differing from other respiratory viruses, SARS-CoV-2 has been circulating in waves but has never fully disappeared during the last 3 years. This has been due to a novel and pandemic virus that differed antigenically enough from other hCoV to encounter an immunologically naïve population. With SARS-CoV-2 becoming endemic and facing substantial herd immunity, it will likely virtually disappear in the summer months as other respiratory viruses.

Several variants have emerged during the last 3 years. Four of them have been designated “variants of concern” by the World Health Organization (WHO). Particularly, the alpha, delta, and omicron variants have quickly replaced previous variants, due to immune escape or higher replication capacity.

Compared to other respiratory viruses, SARS-CoV-2 is transmitted very efficiently between humans. In early stages of the pandemic, SARS-CoV-2 was considered to be transmitted by close contacts, droplets, and fomites; aerosols came into the focus later [15].

When SARS-CoV-2 is inhaled through aerosols, it infects epithelial cells of the upper respiratory mucous membranes. Previous studies indicate that SARS-CoV-2 also enters the human body via the oral cavity and readily replicates there [16]. Viral RNA detected in salivary glands is a result of in situ viral replication and cannot stem from the upper respiratory tract for anatomic reasons. Moreover, cellular SARS-CoV-2 receptors ACE2 and TMPRSS have been detected in oral mucosa, including the tongue, gingival tissue, and salivary glands [7]. Huang et al. confirmed oral SARS-CoV-2 infection by in situ hybridization and susceptibility by RNA single-cell sequencing. Saliva, even from asymptomatic persons, was found to infect culture cells ex vivo [17].

SARS-CoV-2 RNA has been detected in several tissues [18, 19]. Accordingly, various cell types, as respiratory and intestinal can be infected [20, 21]

Sequelae in various organs have been documented, and they increased in reinfected people [22].

Long COVID-19 implies symptoms lasting longer than the acute infection phase of 4 weeks (https://www.rki.de/DE/Content/InfAZ/N/Neuartiges_Coronavirus/Long-COVID/Inhalt-gesamt.html). Main symptoms are fatigue, chest pain, dyspnea, and cough [23]. Objective diagnostic criteria for long COVID-19 are not available, so diagnoses rely mainly on subjective symptoms. Distinct etiologies of long-term sequelae have been established by transcriptome-wide investigation [24]. Clinical signs of SARS-CoV-2 replication in the oral cavity, like discoloration, ulceration, and hemorrhage have been reported in long COVID-19 patients [25]*.*Oral lesions can correlate to other long COVID-19 symptoms and are thus of diagnostic value in routine patient care.

Another COVID-19 complication is multisystem inflammatory syndrome. It can occur in children [26, 27] and adults with divergent pathologies [28, 29].

## SARS-CoV-2 laboratory diagnosis and the importance of the oral cavity in its diagnosis

Most commonly, SARS-CoV-2 is detected in nasopharyngeal swabs by nucleic acid tests, usually real-time PCR. The resulting threshold cycle (Ct) values are frequently translated in viral loads. Viral loads or Ct values have been used as a basis for hygiene management. PCRs cannot differentiate infectious viral particles from non-infectious viral particles or RNA fragments. Infectious viral particles can only be detected in permissive cell culture. Such cell culture systems have been established; they reliably cultivate infectious virus from patient samples [30]. More than 7 days after initial positive PCR, no infectious SARS-CoV-2 can be detected in most patients anymore, even under immune suppression [31]. However, these procedures are too time-consuming for routine diagnostics. Antibodies directed to SARS-CoV-2 are detected with enzyme immune assays and pseudo-neutralization assays. The presence of neutralizing antibodies in vulnerable patients can impact further treatment options.

**Fig. 1:**
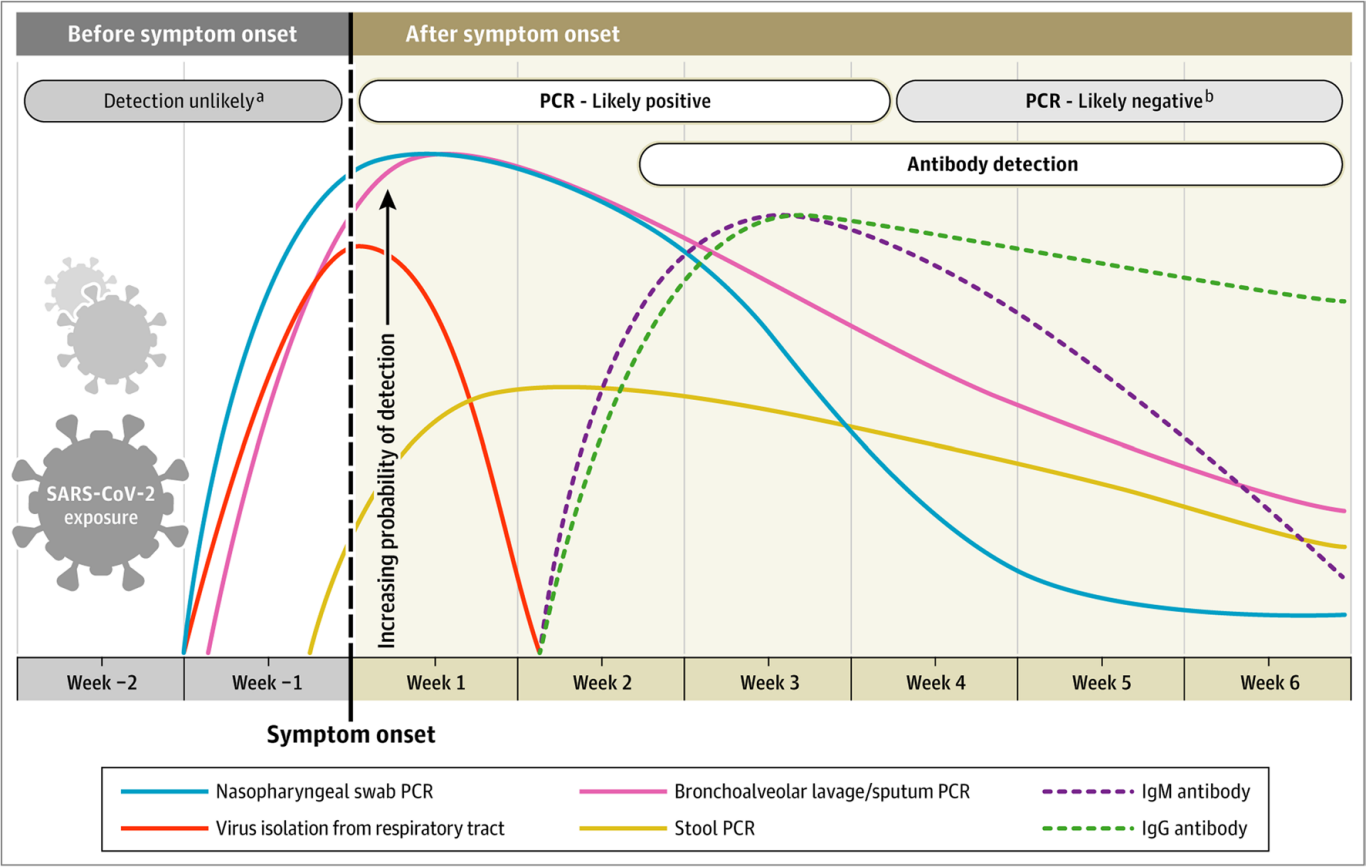
Detection of infectious virus-, RNA, and antibodies during the course of SARS-CoV-2 infection [51]

Figure 1 shows the trajectory of infectious virus and RNA load in different compartments and antibodies.

Around 8–10 days after symptom onset, antibody responses become detectable. In most cases, viral particles are neutralized at this time; thus, infectious virions cannot be detected anymore. At the same time, total RNA begins to decline. Thus, RNA load trajectories and neutralizing antibodies are taken into account to assess if a person is considered infectious. Figure 1 also illustrates that in later infection stages, RNA load can be low or undetectable in nasopharyngeal swabs while still high in bronchoalveolar lavage or sputum. In reinfections, trajectories can vary with lower RNA loads [32] and earlier immune responses.

Since deep nasopharyngeal swabs cause discomfort, alternative sample types have been sought. Saliva and oropharyngeal swabs offer similar sensitivities and easier sampling [33, 34]. This applies to a screening setting [35] and to asymptomatic adults such with mild COVID-19 symptoms [36]. The authors report 4.12 log_10_ copies/mL initial viral load. Personal experience confirms that saliva samples suit well for screening opera staff for SARS-CoV-2 infections (manuscript in preparation).

## Other viruses infecting the oral cavity

### Respiratory viruses

Influenza-, parainfluenza-, metapneumo-, rhino-, adeno-, and respiratory syncytial viruses are also transmitted by aerosols and droplets, as well as direct and indirect contact (fomites). Reproductive numbers *R* lie between 1 and 5. Thus, they are lower than the basic *R* of SARS-CoV-2 at the pandemic start with naïve populations. Influenza viruses, particularly A strains, typically cause more severe illnesses with sudden onset. Respiratory viruses normally replicate only in the epithelial cells of the upper respiratory tract and rarely infect other organs or are detected in blood. However, they may infect lower respiratory tissues in immunocompromised patients, causing atypical pneumonia. With multiplex PCR tests being more frequently employed, respiratory viruses are increasingly detected. Currently, we gain experience what clinical implications that has depending on patient characteristics. Influenza viruses more frequently generalize and cause pneumonia or myocarditis even in persons without pronounced immune deficiency.

### Other viruses affecting the oral mucosa

**Fig. 2:**
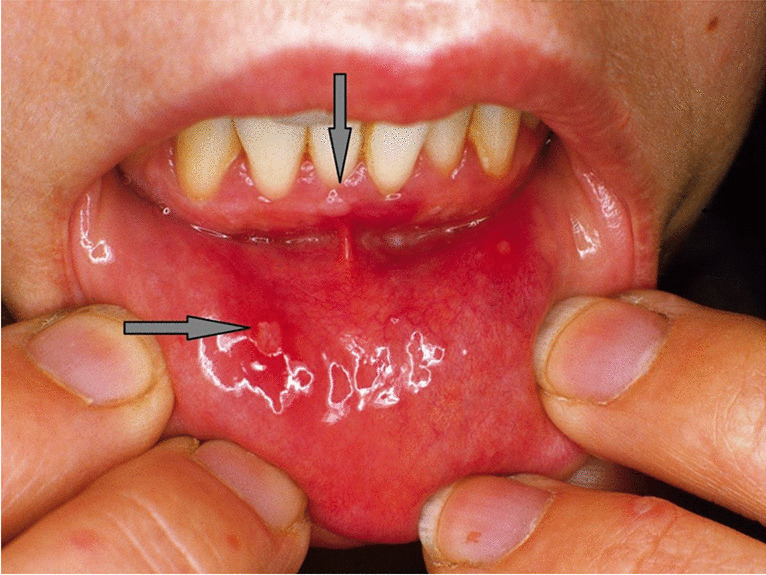
Enterovirus type A infection “hand foot mouth disease” (Altmeyers Enzyklopädie, www.altmeyers.org)

**Fig. 3:**
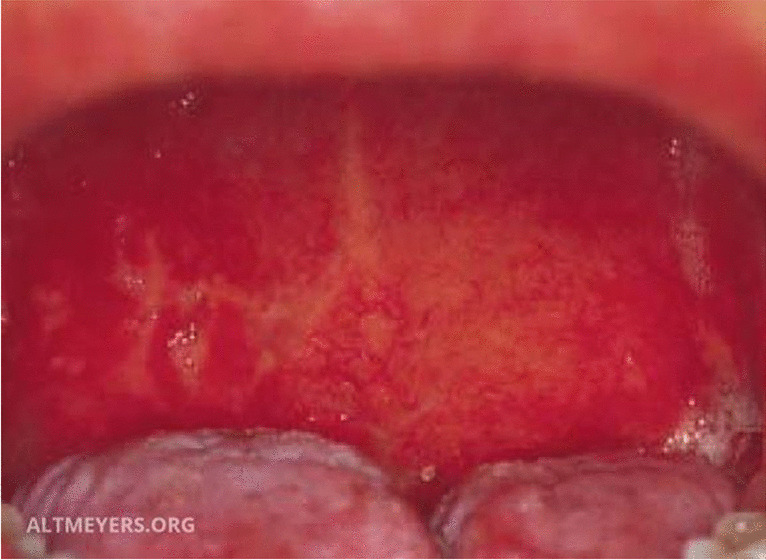
Herpes simplex virus stomatitis (Altmeyers Enzyklopädie, www.altmeyers.org)

Enteroviruses, as well as cytomegalovirus (CMV), Epstein–Barr virus (EBV), herpes simplex virus (HSV), and varicella-zoster virus (VZV), infect epithelial cells of the oral cavity. Unlike than respiratory viruses, they cause more specific lesions like ulcers and blisters (Figs. 2 and 3). The oral cavity is the main entry region of these viruses, from where they spread to other tissues and organs. High viral loads are present in primary infection, e.g., EBV mononucleosis and stomatitis herpetica (Fig. 3). Local EBV reactivations in the oral mucosa are usually asymptomatic in immunocompetent persons and can still infect seronegative persons through infectious droplets.

Measles, mumps, and rubella typically occur in unvaccinated children and young adults. They are highly contagious with R0 reaching 10 in susceptible populations. The best prophylaxis is a double immunization with live attenuated vaccines. If lesions in the oral cavity occur, they are unspecific and hard to interpret (e.g., Koplick spots indicating measles).

Human papillomaviruses (HPV) differ from the above in host infection, where the latter infect chronically, but not necessarily for life. High-risk HPV have been increasingly detected in the oral cavity [37]. Consequently, the incidence for HPV related cancers of the oral cavity rises worldwide, even in younger populations [38]. HPV are transmitted by direct and prolonged contact, e.g., kissing and sexual activities. They persist for several months to years after primary infection. HPV vaccines elicit specific IgG antibodies in saliva and thus can protect individuals from new HPV infections [39]. HPV DNA in saliva could serve as a tumor marker for HPV-induced head and neck cancers [40].

## Diagnosis of viral infections of the oral cavity

Most viruses replicating in the oral cavity do not cause visible lesions. Laboratory diagnosis is important, preferably carried out by real-time PCR. Antigen tests are less sensitive [41, 42] as they do not amplify viral antigenic proteins. Pathognomonic lesion, e.g., Koplick spots, can lead to an immediate diagnosis. Swabs from affected areas offer a good sensitivity. Without obvious lesions, oral rinses or saliva are good alternatives as they come into contact with most of the mucous membrane tissue. Some viruses, e.g., herpesviruses, can replicate independently in different body sites. Thus, detecting CMV in the oral cavity does not establish the diagnosis of a CMV pneumonia but hints at a CMV mucositis. In fact, particularly EBV but also other herpesviruses can be detected in immunocompetent individuals without mucosal abnormalities [43]. On the other hand, a positive PCR detection of respiratory virus in the oral cavity clearly indicates an active infection with viral replication. Influenza/RS viruses were detected at similar rates in saliva and nasopharyngeal aspirates of patients with respiratory infections [44, 45]. Testing of saliva samples, in addition to nasopharyngeal specimen, was reported to detect more respiratory viruses than nasopharyngeal specimen alone [46]. Thus, saliva seems suitable not only for detecting SARS-CoV-2 and influenza viruses but also for other respiratory viruses (adeno-, metapneumo- parainfluenza-, and RS viruses). Being a notorious source of droplets and aerosols, the oral cavity plays an important role in transmitting respiratory viruses.

## General measures against COVID-19 and other infections transmitted via the upper respiratory tract and the oral cavity

Pharmaceutical interventions, e.g., vaccines or antiviral drugs, are distinguished from non-pharmaceutical interventions which consist of contact prevention, i.e., self-isolation and quarantines, contact tracing, triage of patients with respiratory symptoms, and masks.

If used for treatment, antiviral drugs require a virological diagnosis. Alternatively, they can be used for prophylaxis to prevent viral replication.

Vaccines do not directly inhibit viral replication but trigger the host’s immune system to mount an antibody and T cell response against a pathogen. Usually, they are administered as a prophylaxis to prevent or alleviate an infection, but they can also boost the host’s immune response during a chronic infection and thus help treat it (therapeutic vaccination).

Non-pharmaceutical interventions do not require a virological diagnosis, as they work for all viruses. Social distancing limits the spread of every pathogen. Most human pathogenic viruses are transmitted by aerosols and larger droplets. Thus, face masks are effective barriers.

## Virological and clinical effects of mouth rinses

There are two different ways of studying the impact of antiviral mouth rinses:

1. Assay with stable cell culture system whether rinsing reduces infectious viral particles.
2. Assess the effect of mouth rinses in controlled clinical studies.

Numerous antiviral components have inhibited coronavirus replication in high-throughput screening assays [47]. For example, cetylpyridinium chloride (CPC) is active against the hCoV strains OC43, NL63, and MERS. Commercially available mouth rinses inhibited hCoV infectiousness for Huh7 cells by up to 99.9% [48]. Virucidal assays showed that chlorhexidine was effective against influenza- and parainfluenza-, herpes simplex-, and cytomegalovirus in 30 s [49].

A CPC formulation significantly reduced cold symptoms in a randomized placebo-controlled double-blind trial with 94 participants [50]. Three control individuals were tested PCR positive for respiratory viruses, whereas no test person was positive. These encouraging results warrant larger multi-center studies. The effect of preprocedural mouth rinses in a dentist setting is discussed separately in this supplement by J. Weber et al.

## Conclusions

The SARS-CoV-2 pandemic has boosted research in viral transmission and expedited our understanding of viral replication in the oral cavity. Respiratory viruses causing acute infections as well as herpes- and papillomaviruses infecting the host chronically are transmitted through aerosols and droplets. The oral cavity is an important reservoir of many viruses and thus a source of infection. Reducing the pathogens at the site of their multiplication, i.e., in the oral cavity, through the use of antiviral mouth rinses can, at least temporarily, reduce the viral load in aerosols and droplets.

Even though the COVID-19 pandemic caused tremendous suffering, the lessons learned make us much better prepared for future pandemics but also for endemic infectious diseases.

## Data Availability

No specific data other than the cited publications have been used.
